# Impact of the national drug price negotiation policy on the utilization, cost, and accessibility of anticancer medicines in China: A controlled interrupted time series study

**DOI:** 10.7189/jogh.12.11016

**Published:** 2022-12-17

**Authors:** Lele Cai, Tiantian Tao, Hongtao Li, Zhuolin Zhang, Lingli Zhang, Xin Li

**Affiliations:** 1 Department of Pharmaceutical Regulatory Science and Pharmacoeconomics, School of Pharmacy, Nanjing Medical University, Nanjing, China; 2 Department of Health Policy, School of Health Policy and Management, Nanjing Medical University, Nanjing, China; 3 Center for Global Health, School of Public Health, Nanjing Medical University, Nanjing, China

## Abstract

**Background:**

China implemented the national drug price negotiation (NDPN) policy to include 17 innovative anticancer medicines in the national reimbursement drug list in 2018. We aimed to assess the impact of this policy on the utilization, cost, and accessibility of anticancer medicines.

**Methods:**

We obtained monthly medicine procurement data from 1039 hospitals from October 2017 to December 2019. We examined changes in availability, utilization, defined daily dose cost (DDDc), and affordability of the medicines using descriptive statistics and controlled interrupted time series analysis, measuring utilization by defined daily doses (DDDs). Cetuximab and raltitrexed were compared separately for the same indication.

**Results:**

The mean availability of 17 negotiated anticancer medicines was 28.78% after the NDPN, amounting to an increase of 25.22%. The availability increased by 7.88% (95% confidence interval (CI) = 4.31%, 11.45%, *P* < 0.001) immediately and by 1.23% (95% CI = 0.81%, 1.64%, *P* < 0.001) per month after policy implementation. Compared with the control group, the utilization of the medicines increased by 11.44 DDDs (95% CI = 2.42, 20.46, *P* = 0.014) immediately and by 3.54 DDDs (95% CI = 2.47, 4.60, *P* < 0.001) per month after policy implementation, while the DDDc decreased by US$109.09 (95% CI = 68.14, 150.05, *P* < 0.001) immediately and remained stable thereafter. The results on cetuximab and raltitrexed were similar. Availability and utilization differed among regions in east, middle, and west China. Out-of-pocket costs decreased by 17.35 times the catastrophic health expenditures to 1.99 times, but the affordability ratio for 14 negotiated medicines was still greater than 1.

**Conclusions:**

The NDPN policy improved the availability, utilization, and affordability of anticancer medicines. China's experience in NDPN provides a reference for other countries. However, the availability and affordability of anticancer medicines still need further improvement.

Cancer has become a leading cause of death globally, accounting for nearly 10 million deaths, or nearly one in six deaths in 2020 [1]. Among them, lung cancer was the most common cause of cancer death, accounting for 1.80 million. In China, there were 4.57 million new cancer cases, accounting for 23.7% of the cases globally, and three million cancer deaths, accounting for 30% of cancer deaths globally in 2020 [2]. China ranked first in the world in both the number of new cancer cases and cancer deaths, far surpassing other countries in the world. Cancer has become a major problem affecting human health. Simultaneously, cancer treatment expenditures appear to be catastrophic for patients in China [3]. About half of cancer patients borrowed money or went into debt and approximately 10% of cancer patients reported forgoing some medical care because of cost [4]. Many cancer patients cannot afford targeted anticancer medicines – the main cancer treatment [5].

To promote the utilization and accessibility of innovative medicines, improve medical insurance enrolees’ health benefits, and relieve the patients’ financial burden, the Chinese government designed a national drug price negotiation (NDPN) policy in 2016. The main aim of the NDPN between the central government and pharmaceutical enterprises was to reduce the price of innovative medicines with high clinical value. The price of medicines was reduced prior to them being included in the national reimbursement drug list (NRDL), and such medicines were called “national negotiated medicines” [6]. Patients can obtain reimbursement for negotiated medicines, which will improve cost-effectiveness and increase affordability. To ensure the accessibility of negotiated medicines, public hospitals must purchase them via the provincial medicine procurement platform, where the negotiated prices are the maximum ones [7]. For pharmaceutical enterprises, the sales of the negotiated medicine are expected to increase rapidly to achieve a high market share due to being included in the NRDL, which is why they were willing to participate in the negotiations.

In 2016, the Ministry of Health (MoH) conducted the first round of NDPN and successfully negotiated two expensive targeted anticancer medicines, icotinib and gefitinib. Immediately following the 2016 negotiation, the Ministry of Human Resources and Social Security initiated the second round of NDPN in 2017. In the 2017 negotiation, 36 innovative medicines were successfully negotiated – among them,15 were innovative anticancer medicines. The third round of NDPN was held in 2018;17 anticancer medicines were successfully included in the NRDL and the 31 provinces had to ensure that their implementation began at the end of November 2018 [8].

Although China also previously conducted NDPN in 2016 and 2017, the 2018 negotiation was specifically for anticancer medicines and was carried out by China's newly established National Healthcare Security Administration (NHSA). The NHSA incorporated previously separated purchasing power (including price setting, bidding and procurement, and provider payments) to improve social health insurance in a systematic and coordinated way [9]. Since 2018, the NHSA has implemented four rounds of NDPN to introduce 17, 70, 96, and 67 innovative and unaffordable medicines into the NRDL [10].

Previous studies evaluated the impact of the two earlier rounds of NDPN in China [7,10,11]. One study had shown that the second round of negotiation reduced medication prices, increased volumes, and decreased hospital spending on targeted anticancer medications. However, the availability and affordability were not analysed, and the utilization of some medicines showed an unexpected downward trend [7]. Another study found that the second round of national negotiation increased the volume and expenditure of three anticancer drugs and improved their availability in 11 provinces. However, only three medicines were included and the increase in the availability of some medicines was minor [10]. We previously found that the 2017 NDPN improved the utilization and affordability of anticancer medicines in Nanjing [12]; however, our results are not generalizable, as we performed the study in only one Chinese city. We also found a lag time between the release and implementation of the NDPN policy. Previous studies showed the existence of a one-month lag for the 2017 national negotiation [7], and another two-month lag for the 2015 provincial negotiation in Zhejiang Province [13]. The effect of the previous two rounds of NDPN was not fully satisfactory; for example, the increase in availability and utilization did not fulfil expectations, and the aforementioned lag time existed between release and implementation. Meanwhile, as a public strategic buyer, the NHSA collectively managed the selection of NRDL, national price negotiation, and procurement of innovative anticancer medicines [14-16]. However, prior to the establishment of NHSA, drug prices, drug payment standards, insurance listing, drug bidding, and procurement were regulated by different government departments in China [17]. The new centralized NHSA was expected to more quickly increase the availability and utilization of negotiated medicines. Moreover, when designing and implementing the NDPN policy during the 2016 and 2017 negotiations, policymakers could not consider some factors associated with the poor availability of negotiated medicines for cancer patients in public hospitals. For instance, previous studies have suggested that several factors can impede the accessibility of negotiated anticancer medicines due to their relatively high prices. Given the restrictions on the ratio of drug costs to total medical costs, global budget payment systems, and drug zero mark-up policies, Chinese public hospitals generally lack the incentives to provide patients with negotiated anticancer drugs [10]. Thus, the effectiveness of the NDPN policy usually is often questioned.

To solve this problem, the NHSA and other relevant departments have taken a series of complementary measures in the 2018 negotiation to actively improve the accessibility of negotiated medicines. The cost of negotiated medicines has not been covered by the global budget control and was accounted for separately. Local government departments should not hinder the supply and use of negotiated medicines with the excuse of global budget control, the proportion of the cost of medicines in the total cost, and the hospital drug list [18]. Therefore, it is necessary to evaluate whether the improved 2018 negotiations will have the desired effect. To design and evaluate the NDPN policy, governmental decision-makers need to collect scientific evidence on the use of medicines [19]. To our knowledge, there is no previous study on the effects of the 2018 negotiation on 17 anticancer medicines. This study was designed to further investigate whether the NDPN leads to a decrease in cost and a gradual increase in the availability and affordability of anticancer medicines, using nationally representative data.

## METHODS

### Study design

We observed the changes in the availability, utilization, cost, and affordability of 17 innovative anticancer medicines before and after the implementation of the NDPN in November 2018. We collected continuous monthly purchasing data from various hospitals nationwide from October 2017 to December 2019 – ie, analysed the data for the 13 months before the policy implementation and 14 months after the policy implementation.

We used descriptive statistics to summarize the availability and affordability of 17 anticancer medicines before and after the policy implementation. September 2018 and December 2019 were selected as the time points for evaluating the availability before and after the implementation of the policy.

We examined changes in availability, utilization, and cost using controlled interrupted time series (ITS) analysis. The policy intervention group was innovative anticancer medicines that were included in the NRDL through negotiation in 2018. Because the five negotiated medicines, azacitidine, anlotinib, ceritinib, vimofenib, and isazomib, were procured less than 12 months before the policy intervention and did not have sufficient observation points, we did not include them in the ITS analysis, leaving the 12 negotiated medicines as the intervention group. The control group was four anticancer medicines that were not involved in the NDPN during the entire study period and included ruxolitinib, raltitrexed, metuximab, and paclitaxel liposome. Moreover, cetuximab and raltitrexed were selected for ITS analysis because both had indications for colorectal cancer; one was included in the NRDL and the other was not. There were few differences in the indications between the intervention group medications and control group medications. In the intervention group, most medications were indicated for the common solid tumours and leukaemia, such as non-small cell lung cancer (n = 5), colorectal cancer (n = 2), kidney cancer (n = 3), and haematological malignancies (non-solid tumours, n = 3). As for the control group, four medications were indicated for solid tumours including lung cancer, colorectal cancer, breast cancer, and liver cancer, and one medication was for myelofibrosis therapies (non-solid tumours).

### Data sources and outcome measures

Data were obtained from the China Medicine Economic Information (CMEI) database. The database aggregates procurement data from more than 1500 hospitals nationwide. These hospitals covered 31 provinces, autonomous regions, and municipalities throughout the eastern, middle, and western regions of China. These were mainly tertiary and secondary public hospitals, including both general and specialized hospitals. We included 1039 hospitals that continuously reported monthly the anticancer medicine procurement data, including 740 tertiary hospitals and 299 secondary and lower hospitals, which covered over 29.04% of tertiary and 3.32% of secondary hospitals in China in 2018, respectively [20].

#### Availability

We evaluated the availability of anticancer medicines by the standardized survey method issued by World Health Organization and Health Action International (WHO/HAI). We calculated the availability separately for tertiary and secondary hospitals:

*Availability =* (*the number of hospitals that procured the medicine/the number of hospitals*) × 100%

The following criteria were used to describe the level of availability [21]:

− Absent: 0%, the negotiated anticancer medicine was not available in any hospital;
− Very low: <30%, the negotiated anticancer medicine was available hardly in hospitals;
− Low: 30-50%, the negotiated anticancer medicine was available in few hospitals;
− Fairly high: 50-80%, the negotiated anticancer medicine was available in many hospitals;
− High: >80%, the negotiated anticancer medicine had good availability and was available in most hospitals. We also calculated the availability of different regions, dividing China into three regions: east, middle and west.

#### Utilization

Utilization – defined as the average hospital utilization per month for each anticancer medicine – was calculated for each region. The medicine utilization was measured by defined daily doses (DDDs):

*DDDs* = *total quantity procured/DDD*

#### Cost

We measured the treatment cost of medicine using deﬁned daily dose cost (DDDc). A lower DDDc indicated that the medicine was cheaper. Costs were converted into and reported in US dollars (US$) based on the average exchange rate (US$1 = CNY6.6174 in 2018) [22].

#### Affordability

The measure of affordability is the ratio of annual out-of-pocket (OOP) cost for medicines and catastrophic health expenditures. If the ratio is less than 1, the medicine is affordable; if the ratio is greater than 1, the medicine is unaffordable and pushes the patient into poverty. Catastrophic health expenditures are defined as health expenditures that exceed a certain percentage of the income remaining after survival needs are met [23]. The commonly used percentage in China is 40% [24-26]. The income remaining after survival needs is based on the average annual consumption expenditure of Chinese residents in 2018 [27]. The reimbursement rate for anticancer medicines included in the NRDL is 70% [28] and the OOP expenditure is 30% of the treatment cost. For medicines not included in the NRDL, patients pay the full cost of OOP. We calculated the OPP cost of the medicine based on the duration of treatment within one year in the standard treatment protocol [29]. In this study, we assessed the annual duration of the anticancer medicine based on the median progression-free survival (mPFS). If mPFS was more than one year, the duration of therapy was set to one year, and if mPFS was less than one year, mPFS was the annual duration of therapy. For the medicines lacking mPFS, the duration of therapy was assessed according to treatment guidelines and relevant clinical trials.

### Data analysis

We used single-group and multiple-group ITS analysis to assess the impact of national negotiation policies on the accessibility of anticancer medicines. The single-group ITS analysis was conducted using the following regression model:

*Y_t_* = *β_0_ + β_1_T_t_ + β_2_Xt + β_3_X_t_T_t_ + ε_t_*

*β_0_* represents the intercept, *β_1_* is the slope before the intervention, *β_2_* represents the change in the level that occurs immediately following the introduction of the intervention (compared with the counterfactual), *β_3_* represents the difference between preintervention and postintervention slopes of the outcome.

We conducted the multiple groups ITS analysis using the following regression model:

*Y_t_ = β_0_ + β_1_T_t_ + β_2_X_t_ + β_3_X_t_T_t_ + β_4_Z + β_5_ZT_t_ + β_6_ZX_t_ + β_7_ZX_t_T_t_ + ε_t_*

*β_0_* to *β_3_* represent the control group (similar to the single-group ITS model), while *β_4_* to *β_7_* represent the treatment group. *β_4_* represents the difference in the level between treatment and control before the intervention, *β_5_* the difference in the slope between treatment and control before the intervention, *β_6_* the difference in the level between treatment and control immediately following the intervention initiation and *β_7_* the difference between treatment and control in the slope after initiation of the intervention compared with preintervention.

In the above two models, *Y_t_* is the aggregated outcome variable measured at time point t of each month, *T_t_* is the time since the start of the study, *X_t_* is a dummy variable representing the intervention, and *Z* indicates the treatment status (*Z* = 1 for the treatment group and *Z* = 0 for the control group). *X_t_T_t_*, *ZT_t_*, *ZX_t_*, and *ZX_t_T_t_* are interaction terms [30].

We chose the Newey model to estimate the coefficients by ordinary least squares (OLS) regression and produced Newey-West standard errors to handle autocorrelation alongside possible heteroskedasticity [31,32]. We ran all the models using the statistical software Stata/MP V.16.0 (StataCorp).

## RESULTS

### Characteristics of negotiated anticancer medicines

Among the 17 negotiated anticancer medicines, three were launched in China in 2018, eight were launched in 2017, and six were launched before 2017 (**Table 1**). Among them, anrotinib and ceritinib were launched in May 2018 and quickly entered the NRDL at the end of September 2018. Regarding indications, there were up to five medicines for non-small cell lung cancer (NSCLC), namely afatinib, anlotinib, oseltinib, crizotinib, and ceritinib. Regarding the target, the EGFR target had the greatest number of medicines at six. There were 13 targeted anticancer medicines and only four were chemotherapy medicines.

**Table 1:** Characteristics of anticancer medicine in the NDPN policy intervention group and control group

Generic name	Dosage form	Launch time in China	Marketing authorization holder	Indications listed in NRDL	Target
**Policy intervention group**					
Afatinib	Tablets	February 2017	Boehringer Ingelheim	Non-small cell lung cancer	EGFR
Axitinib	Tablets	April 2015	Pfizer	Renal cell carcinoma	VEGFR
Azacitidine	Injections	April 2017	Celgene	Myelodysplastic syndrome / chronic myelomonocytic leukemia / acute myeloid leukemia	-
Anlotinib	Capsules	May 2018	Chia Tai Tianqing	Non-small cell lung cancer	EGFR
Octreotide	Microsphere injections	August 2003	Novartis	Gastrointestinal pancreatic endocrine tumor	-
Osimertinib	Tablets	March 2017	AstraZeneca	Non-small cell lung cancer	EGFR
Crizotinib	Capsules	January 2013	Pfizer	Non-small cell lung cancer	EGFR
Nilotinib	Capsules	July 2009	Novartis	Chronic myeloid leukemia	BCR-ABL
Pegaspargase	Injections	December 2017	Jiangsu Hengrui	Childhood acute lymphoblastic leukemia	-
Pazopanib	Tablets	March 2017	Novartis	Renal cell carcinoma	VEGFR
Regorafenib	Tablets	March 2017	Bayer	Hepatocellular carcinoma/colorectal cancer/gastrointestinal stromal tumor	VEGFR, KIT, RET
Ceritinib	Capsules	May 2018	Novartis	Non-small cell lung cancer	EGFR
Sunitinib	Capsules	October 2007	Pfizer	Renal cell carcinoma/gastrointestinal stromal tumor/pancreatic neuroendocrine tumor	VEGFR, PDGFR, KIT, FLT, RET
Vemurafenib	Tablets	March 2017	Roche	Melanoma	BRAF
Cetuximab	Injections	December 2015	Merck	Colorectal cancer	EGFR
Ibrutinib	Capsules	August 2017	Janssen	Mantle cell lymphoma/chronic lymphocytic leukemia/small lymphocytic lymphoma	BTK
Ixazomib	Capsules	April 2018	Takeda	Multiple myeloma	-
**Control group**					
Ruxolitinib	Tablets	March 2017	Novartis	Myelofibrosis	JAK
Raltitrexed	Injections	September 2009	Chia Tai Tianqing	Colorectal cancer	-
Metuximab	Injections	May 2005	Chengdu Huashen	Liver cancer	VEGFR
Paclitaxel liposome	Injections	January 2003	Nanjing Luye	Ovarian cancer, breast cancer, non-small cell lung cancer	-

### Impact of the NDPN policy on availability

The availability of all 17 negotiated anticancer medicines increased by an average of 25.22% after the implementation of the NDPN policy (**Table 2**). The increase in the availability in the control group (3.72%) was much lower than that in the intervention group. The mean availability of 17 negotiated anticancer medicines was 28.78% after the NDPN. Both before and after the negotiation, the availability of anticancer medicines in tertiary hospitals was higher than that in secondary and lower hospitals. After the implementation of the NDPN policy, the average availability of 17 negotiated anticancer medicines in tertiary hospitals was 36.49%, compared to only 9.7% in secondary and lower hospitals. The top three medicines in terms of availability after the NDPN were anrotinib (52.07%), ositinib (48.99%), cetuximab (39.65%), while vimofenib had the lowest availability (10.11%). The TOP 3 medicines with an absolute increase in availability were anrotinib (49.57%), ositinib (46.39%), and crizotinib (34.46%), all three of which were for the treatment of NSCLC. A different availability has been noticed with respect to regions: it was the highest for the eastern region, followed by the middle and western, both before and after the NDPN (Table S1 in the **Online Supplementary Document**).

**Table 2:** Availability of anticancer medicines before and after the national drug price negotiation policy

Generic name	Availability before NDPN (%)			Availability after NDPN (%)			Difference in total availability (%)	Ratio of changes in total availability*
	**Tertiary hospital**	**Secondary and lower hospital**	**Total hospital**	**Tertiary hospital**	**Secondary and lower hospital**	**Total hospital**		
**Policy intervention group**								
Afatinib	1.08	0.33	0.87	42.57	12.37	33.88	33.01	37.94
Axitinib	0.68	0.00	0.48	28.11	6.02	21.75	21.27	44.31
Azacitidine	0.41	0.00	0.29	37.43	8.03	28.97	28.68	98.90
Anlotinib	3.24	0.67	2.50	61.08	29.77	52.07	49.57	19.83
Octreotide	10.14	1.00	7.51	29.19	4.35	22.04	14.53	1.93
Osimertinib	3.24	1.00	2.60	58.78	24.75	48.99	46.39	17.84
Crizotinib	2.57	0.00	1.83	45.81	12.71	36.28	34.46	18.83
Nilotinib	13.11	1.00	9.62	31.22	6.02	23.97	14.34	1.49
Pegaspargase	21.08	3.68	16.07	38.11	6.69	29.07	12.99	0.81
Pazopanib	1.08	0.00	0.77	21.35	3.34	16.17	15.40	20.00
Regorafenib	1.62	0.00	1.15	40.95	9.70	31.95	30.80	26.78
Ceritinib	0.00	0.00	0.00	22.30	1.67	16.36	16.36	-
Sunitinib	5.41	0.00	3.85	36.49	8.70	28.49	24.64	6.40
Vemurafenib	0.00	0.00	0.00	13.65	1.34	10.11	10.11	-
Cetuximab	14.86	2.68	11.36	48.51	17.73	39.65	28.30	2.49
Ibrutinib	1.62	0.00	1.15	34.32	8.03	26.76	25.61	22.27
Ixazomib	0.68	0.00	0.48	30.41	3.68	22.71	22.23	46.31
Mean (SD)	4.75 (6.23)	0.61 (1.06)	3.56 (4.72)	36.49 (12.60)	9.70 (7.88)	28.78 (11.13)	25.22 (11.32)	24.41 (25.53)
**Control group**								
Ruxolitinib	1.08	0.00	0.77	2.97	0.33	2.21	1.44	2.87
Raltitrexed	37.57	13.71	30.70	45.81	19.40	38.21	7.51	1.24
Metuximab	14.00	0.00	0.10	0.41	0.00	0.29	0.19	2.90
Paclitaxel liposome	42.70	13.04	34.17	48.65	18.39	39.94	5.77	1.17
Mean (SD)	23.84 (19.66)	6.69 (7.73)	16.44 (18.53)	24.46 (26.34)	9.53 (10.82)	20.16 (21.86)	3.73 (3.47)	2.05 (0.97)

NDPN – national drug price negotiation, SD – standard deviation

*The symbol “-” means that the availability before NDPN is zero and cannot be calculated.

**Figure 1:**
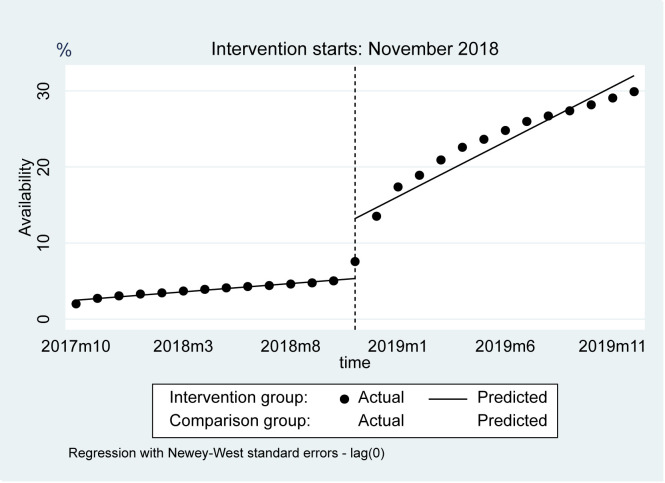
Observed and predicted availability of 2018 negotiated medications and comparison group.

The availability of the intervention group increased by 7.88% (95% confidence interval (CI) = 4.31%, 11.45%, *P* < 0.001), while it decreased for the comparison group by 0.98% (95% CI = 0.13%, 1.84%, *P* = 0.026) in the first month after policy implementation (**Figure 1** and **Table 3**). After the implementation of the NDPN policy, the trend in the intervention group increased by 1.23% (95% CI = 0.81%, 1.64%, *P* < 0.001) compared with that before the implementation of the policy. The intervention group had a higher trend than the control group after the implementation of the NDPN policy (1.19%; 95% CI = 0.79%, 1.59%, *P* < 0.001).

**Table 3:** Changes in levels and trends of availability, utilization, and cost for the intervention group and control group

Category	Level and trend				Trend estimation after NDPN policy intervention
	**Baseline level**	**Baseline trend**	**Level change immediately after NDPN Policy intervention**	**Trend change after NDPN policy intervention**	
**Availability (%)**					
Intervention group	2.26 (95% CI = 1.92, 2.61, *P *< 0.001)	0.22 (95% CI = 0.18, 0.26, *P *< 0.001)	7.88 (95% CI = 4.31, 11.45, *P *< 0.001)	1.23 (95% CI = 0.81, 1.64, *P *< 0.001)	1.45 (95% CI = 1.03, 1.86, *P *< 0.001)
Comparison group	10.14 (95% CI = 8.81, 11.48, *P *< 0.001)	0.56 (95% CI = 0.41, 0.71, *P *< 0.001)	-0.98 (95% CI = -1.84, -0.13, *P* = 0.026)	-0.31 (95% CI = -0.46, -0.16, *P *< 0.001)	0.25 (95% CI = 0.24, 0.27, *P *< 0.001)
Differences between the intervention and control group	-7.88 (95% CI = -9.22, -6.54, *P *< 0.001)	-0.34 (95% CI = -0.49, -0.19, *P *< 0.001)	8.86 (95% CI = 5.29, 12.44, *P *< 0.001)	1.53 (95% CI = 1.10, 1.96, *P *< 0.001)	1.19(95% CI = 0.79, 1.59, *P *< 0.001)
**Utilization (DDDs)**					
Intervention group	3.48 (95% CI = 3.01, 3.96, *P *< 0.001)	0.07 (95% CI = 0.01, 0.12, *P *= 0.018)	12.66 (95% CI = 5.83, 19.48, *P* = 0.001)	3.10 (95% CI = 2.21, 3.99, *P *< 0.001)	3.17 (95% CI = 2.28, 4.05, *P *< 0.001)
Comparison group	11.83 (95% CI = 9.70, 13.96, *P *< 0.001)	0.60 (95% CI = 0.21, 0.99, *P* = 0.004)	1.22 (95% CI = -5.05, 7.49, *P* = 0.691)	-0.44 (95% CI = -1.08, 0.20, *P* = 0.172)	0.17 (95% CI = -0.35, 0.68, *P* = 0.508)
Differences between the intervention and control group	-8.35 (95% CI = -10.47, -6.22, *P *< 0.001)	-0.54 (95% CI = -0.92, -0.16, *P* = 0.007)	11.44 (95% CI = 2.42, 20.46, *P* = 0.014)	3.54(95% CI = 2.47, 4.60, *P *< 0.001)	3.00 (95% CI = 2.01, 3.99, *P *< 0.001)
**Cost (DDDc in US$)**					
Intervention group	251.84 (95% CI = 235.02, 268.67, *P *< 0.001)	-2.74 (95% CI = -6.12. 0.64, *P* = 0.107)	-124.50 (95% CI = -155.87, -93.13, *P* < 0.001)	2.80 (95% CI = -0.58, 6.18, *P* = 0.100)	0.06 (95% CI = -0.06, 0.18, *P* = 0.282)
Comparison group	1308.80 (95% CI = 1301.46, 1316.14, *P *< 0.001)	-0.24 (95% CI = -1.82. 1.33, *P* = 0.752)	-15.41 (95% CI = -43.47, 12.66, *P* = 0.268)	0.33 (95% CI = -3.67, 4.33, *P* = 0.866)	0.09 (95% CI = -3.59, 3.76, *P* = 0.961)
Differences between the intervention and control group	-1056.96 (95% CI = -1074.82, -1039.10, *P *< 0.001)	-2.49 (95% CI = -6.12. 1.13, *P* = 0.173)	-109.09 (95% CI = -150.05, -68.14, *P *< 0.001)	2.47 (-2.63, 7.56, *P* = 0.334)	-0.03 (95% CI = -3.60, 3.55, *P* = 0.989)

NDPN – national drug price negotiation, DDDs – defined daily doses; DDDc – defined daily dose cost, CI – confidence interval

**Figure 2:**
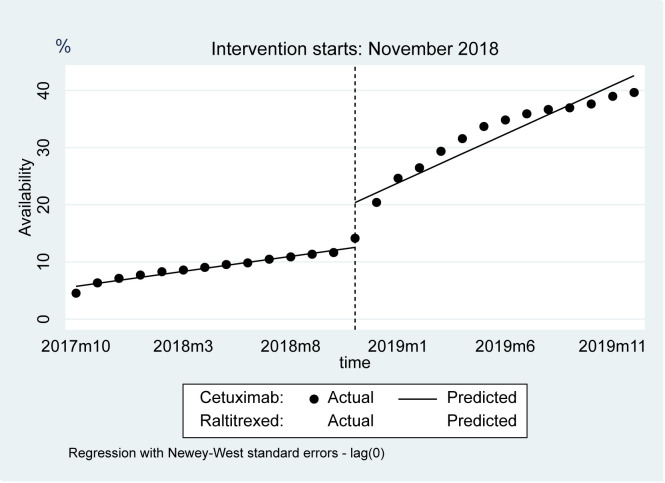
Observed and predicted availability of cetuximab and raltitrexed.

**Figure 2** and **Table 4** showed the changes in the availability of cetuximab and raltitrexed before and after the implementation of the NDPN policy, which were similar to the changes in all negotiated anticancer medicines groups and the control group. Immediately after the implementation of the NDPN policy, the level of cetuximab increased by 7.86% (95% CI = 3.81%, 11.90%, *P* = 0.001), while raltitrexed decreased by 2.50% (95% CI = 0.26%, 4.73%, *P* = 0.030). The trend estimation of cetuximab was higher than that of raltitrexed after the NDPN (1.20%; 95% CI = 0.74%, 1.67%, *P* < 0.001)).

**Table 4:** Changes in levels and trends of availability, utilization, and cost for cetuximab and raltitrexed

Category	Level and trend				Trend estimation after NDPN policy intervention
	**Baseline level**	**Baseline trend**	**Level change immediately after NDPN policy intervention**	**Trend change after NDPN policy intervention**	
**Availability**					
Cetuximab	5.21 (95% CI = 4.31, 6.10, *P *< 0.001)	0.52 (95% CI = 0.43, 0.62, *P *< 0.001)	7.86 (95% CI = 3.81, 11.90, *P* = 0.001)	1.18 (95% CI = 0.70, 1.67, *P *< 0.001)	1.71 (95% CI = 1.23, 2.18, *P *< 0.001)
Raltitrexed	17.38 (95% CI = 13.75, 21.01, *P *< 0.001)	1.21 (95% CI = 0.81, 1.61, *P *< 0.001)	-2.50 (95% CI = -4.73, -0.26, *P* = 0.030)	-0.70 (95% CI = -1.10, -0.30, *P* = 0.001)	0.51 (95% CI = 0.48, 0.53, *P *< 0.001)
Differences between cetuximab and raltitrexed	-12.17 (95% CI = -15.81, -8.53, *P *< 0.001)	-0.69 (95% CI = -1.09, -0.26, *P* = 0.001)	10.36 (95% CI = 5.86, 14.85, *P *< 0.001)	1.89 (95% CI = 1.27, 2.50, *P *< 0.001)	1.20 (95% CI = 0.74, 1.67, *P *< 0.001)
**Utilization (DDDs)**					
Cetuximab	3.28 (95% CI = 2.77, 3.78, *P *< 0.001)	0.10 (95% CI = 0.03, 0.16, *P* = 0.003)	9.15 (95% CI = 4.33, 13.97, 0.001)	1.71 (95% CI = 1.10, 2.31, *P *< 0.001)	1.81 (95% CI = 1.21, 2.41, *P *< 0.001)
Raltitrexed	35.77 (95% CI = 28.61, 42.92, *P *< 0.001)	2.08 (95% CI = 0.72, 3.44, *P* = 0.004)	4.78 (95% CI = -17.03, 26.59, *P* = 0.655)	-1.52 (95% CI = -3.75, 0.71, *P* = 0.173)	0.56 (95% CI = -1.21, 2.33, *P* = 0.517)
Differences between cetuximab and raltitrexed	-32.49 (95% CI = -39.47, -25.51, *P *< 0.001)	-1.98 (95% CI = -3.30, -0.65, *P* = 0.004)	4.37 (95% CI = -17.36,36.10, *P* = 0.687)	3.23 (95% CI = 0.98, 5.48, *P* = 0.006)	1.25 (95% CI = -0.57, 3.06, *P* = 0.174)
**Cost (DDDc in US$)**					
Cetuximab	726.68 (95% CI = 686.68, 766.68, *P *< 0.001)	-9.34 (95% CI = -16.39, -2.29, *P* = 0.012)	-368.17 (95% CI = -431.05, -305.30, *P *< 0.001)	9.28 (95% CI = 2.22, 16.33, *P* = 0.012)	-0.06 (95% CI = -0.34, 0.21, *P* = 0.634)
Raltitrexed	54.14 (95% CI = 53.90, 54.39, *P *< 0.001)	-0.07 (95% CI = -0.10, -0.05, *P *< 0.001)	-0.03 (95% CI = -0.64, 0.57, *P* = 0.908)	-0.04 (95% CI = -0.19, 0.10, *P* = 0.543)	-0.11 (95% CI = -0.26, 0.03, *P* = 0.107)
Differences between cetuximab and raltitrexed	672.53 (95% CI = 633.61, 711.46, *P *< 0.001)	-9.27 (95% CI = -16.13, -2.41, *P* = 0.009)	-368.14 (95% CI = -429.32, -306.96, *P *< 0.001)	9.32 (95% CI = 2.46, 16.19, *P* = 0.009)	0.05 (95% CI = -0.25, 0.36, *P* = 0.735)

NDPN – national drug price negotiation, DDDs – defined daily doses, DDDc – defined daily dose cost, CI – confidence interval

### Impact of the national drug price negotiation policy on utilization

**Figure 3:**
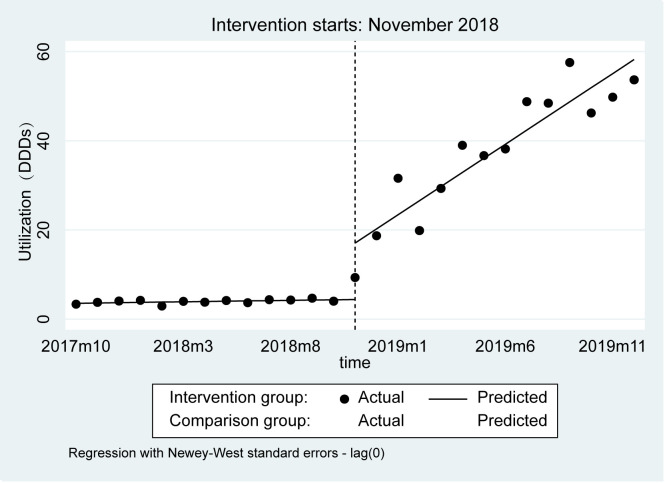
Observed and predicted utilization of 2018 negotiated medications and comparison group.

**Figure 3** and **Table 3** reflected the changes in utilization in the intervention and control groups after the implementation of the NDPN. Both the immediate intervention effect and the effect over time of the intervention group were different from those of the control group after the NDPN policy implementation. In November 2018, the first month after the policy implementation, the average utilization in the intervention group increased rapidly by 11.44 DDDs (95% CI = 2.42, 20.46, *P* = 0.014) compared to the control group. There were differences in the post-intervention trend between the intervention and control groups (3.54; 95% CI = 2.47, 4.60, *P* < 0.001). There was no change in the control group in terms of post-intervention trends, while the average utilization increased by 3.17 DDDs (95% CI = 2.28, 4.05, *P* < 0.001) per month in the intervention group. The utilization changes of 12 negotiated anticancer medicines before and after the NDPN are detailed in Figure S1 in the **Online Supplementary Document**. The monthly utilization nationwide and by region are shown in Figure S2 in the **Online Supplementary Document****;** the highest utilization was in the east and the lowest was in the west.

**Figure 4:**
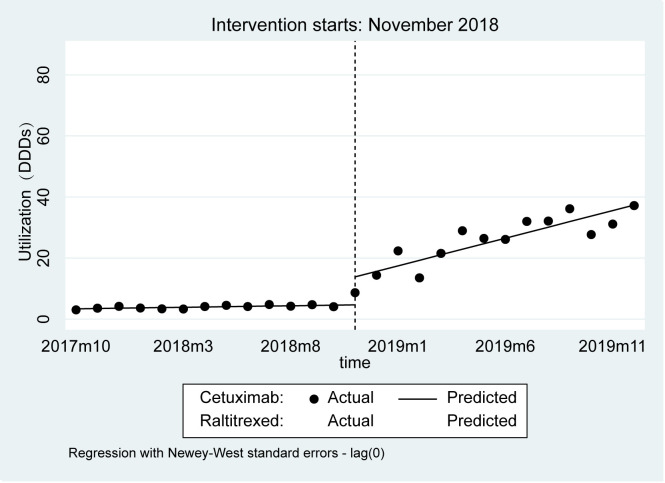
Observed and predicted utilization of cetuximab and raltitrexed.

Both cetuximab and raltitrexed were intended for colorectal cancer, with the former being the 2018 negotiated medicine and the latter not. There were differences in trend changes between cetuximab and raltitrexed after the intervention (3.23; 95% CI = 0.98, 5.48, *P* = 0.006) (**Figure 4** and **Table 4**). In the post-intervention effect, the average hospital utilization of cetuximab increased by 1.81 DDDs (95% CI = 1.21, 2.41, *P* < 0.001) per month, while the change in raltitrexed was 0.56 DDDs (95% CI = -1.21, 2.33, *P* = 0.517).

### Impact of the national drug price negotiation policy on cost

**Figure 5:**
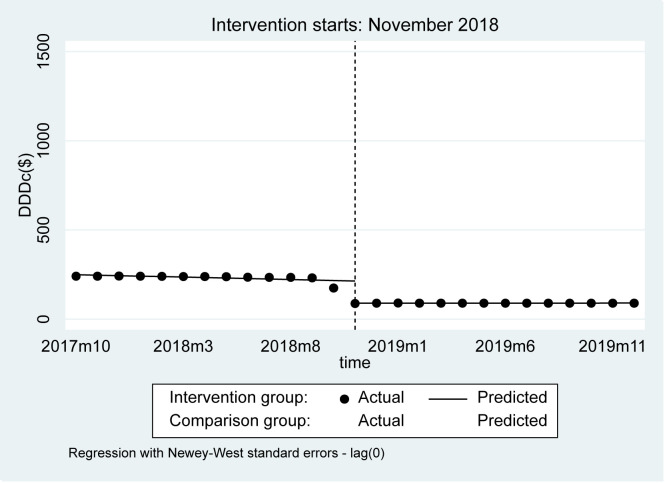
Observed and predicted DDDc of 2018 negotiated medications and comparison group.

The DDDc of the negotiated anticancer medicines decreased by US$124.50 (95% CI = 93.13, 155.87, *P* < 0.001) in the first month immediately after the policy was implemented, while it decreased in the control group by US$15.41 (95% CI = -12.66, 43.47, *P* = 0.268) (**Figure 5** and **Table 3**). Regarding post-intervention trends, the DDDc remained stable in both the intervention and control groups. The cost changes of 12 negotiated anticancer medicines before and after the NDPN is detailed in Figure S3 in the **Online Supplementary Document**.

**Figure 6:**
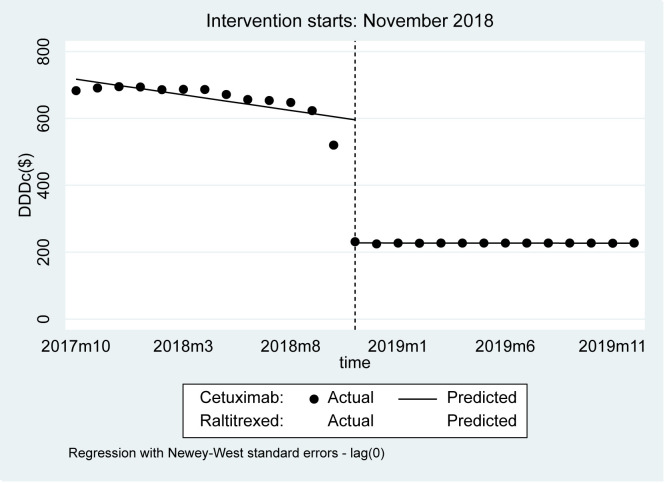
Observed and predicted DDDc of cetuximab and raltitrexed.

The DDDc of cetuximab decreased by US$368.17 (95% CI = 305.30, 431.05, *P* < 0.001) immediately after the NDPN, and remained essentially stable thereafter (**Figure 6** and **Table 4**). In contrast, the DDDc of raltitrexed was essentially unchanged both before and after the NDPN.

### Impact of the national drug price negotiation policy on affordability

The annual OOP cost and affordability for the 17 negotiated anticancer medicines before and after NDPN is shown in **Table 5**. To calculate the cost, we first collected the annual duration of treatment for each medicine. The average annual OOP cost of 17 anticancer medicines was as high as US$44 715.90 before the NDPN. However, the average annual OOP cost was only US$5131.17 after the NDPN, a reduction of 88.52%. The annual OOP cost decreased from 17.35 times the catastrophic health expenditures to 1.99 times, ie, the affordability of these anticancer medicines was improved dramatically. The affordability for anlotinib, pegaspargase, and sunitinib was good, with their annual OOP cost below catastrophic health expenditures. The least affordable medicine was ibrutinib, with the OOP cost up to 4.85 times the catastrophic health expenditure. The affordability ratios for most other negotiated anticancer medicines were about 1-4.

**Table 5:** Affordability of 17 anticancer medicines before and after the national drug price negotiation policy

Generic name	Annual duration of treatment (month)	Before NDPN		After NDPN	
		**Annual cost (US$)**	**Affordability ratio**	**Annual cost (US$)**	**Affordability ratio**
Afatinib	11.0 [33]	16 406.75	6.36	2992.11	1.16
Axitinib	8.3 [34]	53 281.35	20.67	4673.41	1.81
Azacitidine	12.0 [35]	42 484.59	16.48	5122.43	1.99
Anlotinib	5.4 [36]	14 460.06	5.61	2384.44	0.93
Octreotide	12.0 [37]	17 497.51	6.79	3155.32	1.22
Osimertinib	8.2 [38]	65 427.51	25.38	5687.73	2.21
Crizotinib	7.7 [39]	62 275.82	24.16	5445.64	2.11
Nilotinib	12.0 [40]	33 094.57	12.84	3134.06	1.22
Pegaspargase	12.0 [41]	11 992.63	4.65	2161.57	0.84
Pazopanib	9.2 [42]	32 615.83	12.65	3403.39	1.32
Regorafenib	4.8 [43]	21 934.90	8.51	3582.70	1.39
Ceritinib	5.4 [44]	36 721.37	14.25	4362.50	1.69
Sunitinib	5.6 [45]	22 899.63	8.88	2274.73	0.88
Vemurafenib	6.9 [46]	52 051.86	20.19	8408.38	3.26
Cetuximab	9.2 [47]	107 835.71	41.83	9880.70	3.83
Ibrutinib	12.0 [48]	119 140.45	46.22	12 509.75	4.85
Ixazomib	12.0 [49]	50 049.87	19.42	8050.96	3.12
Mean (SD)		44 715.90 (30913.02)	17.35 (11.99)	5131.17 (2956.45)	1.99 (1.15)

NDPN – national drug price negotiation, SD – standard deviation

## DISCUSSION

We found that the availability and utilization of anticancer medicines negotiated in 2018 increased, the cost of treatment decreased, and the affordability was improved. However, the availability and affordability of anticancer medicines remain low.

We identified that the availability of anticancer medicines increased after the NDPN policy. Their availability was very low before the NDPN because most of them had been launched relatively recently. Although the availability increased, it was still low, with less than 30% of hospitals on average being able to provide these medicines. The availability of these medicines was much lower (particularly in secondary and lower hospitals), so there was still a need to further promote their availability. In fact, to further improve the availability of negotiated medicine, the NHSA and the National Health Commission of China implemented a “dual-channel” management policy for negotiated medicines in 2021 [50]. Patients can obtain negotiated anticancer medicines at community pharmacies and hospital pharmacies after visiting public hospitals, and patients are reimbursed for obtaining medicines in both channels. The effectiveness of these complementary measures still needs to be studied. An interesting finding was that several anticancer medicines, such as axitinib and crizotinib, were absent in secondary and lower hospitals before NDPN and became available after it was implemented. This could have caused differences in the treatment plans due to a lack of experience, which could have led to inequality across hospitals. In China, the availability of anticancer medicines was lower in secondary hospitals than in tertiary hospitals, which cancer patients usually visit for their higher health care quality. Therefore, there is a need for training and guidance on the use of these anticancer medicines in secondary hospitals, as well as enhanced monitoring of their use.

This study showed the NDPN policy was highly effective in promoting increased utilization and lower costs of anticancer medicines. The immediate intervention effect was obvious, with an increase in the utilization of anticancer medicines in the first month after the policy implementation. Meanwhile, the effect of policy intervention over time has also been maintained, with the utilization of negotiated anticancer medicines showing an increasing trend in the year after its implementation. This indicates that the negotiation policy benefited many patients. There were two reasons why the manufacturers of these anticancer medicines were willing to reduce their prices and participant in the NDPN. While the national basic medical insurance coverage has reached 95% [51] and the NHSA has strong bargaining power as the largest payer of medical expenditures, China has accelerated the speed of review and approval of new drugs, leading to an increase in the number of drugs on the market and more intense competition. For example, six of the 17 negotiated anticancer medicines were for the treatment of NSCLC and various manufacturers were willing to reduce prices in the hope of gaining an advantageous position in the competition. With the price reduction, patients who could not afford such anticancer medicines can now access them.

We found that affordability was improved through the NDPN policy. Our data were collected through December 2019, after which COVID-19 started to dramatically impact China. The COVID-19 pandemic made it harder for patients to go to the hospital due to the societal lockdown. Yet, the improvement in affordability was mainly due to the reduction in OOP costs for these anticancer medicines, including the reduction in prices and OOP ratios after inclusion in the NRDL. Based on our observations, the NDPN policy is still in effect, and the prices of these medicines are stable below the negotiated prices and remain in the NRDL. Although our study was conducted prior to the COVID-19 pandemic, we believe the substantial improvement in affordability was sustained during the pandemic. However, the costs of many anticancer medicines continued to exceed catastrophic health expenditures for Chinese patients. The affordability of these anticancer medicines needs to be further improved. Innovative anticancer medicines have better clinical outcomes, but often mean high prices and poor affordability. Many countries face the problem of balancing balance the innovation, clinical outcomes, and affordability of anticancer medicines [52-54]. Some recommendations for tackling this issue exist. First, the price of the medicine can be evaluated through health technology assessment [19,55]. Second, the fairness and transparency of the negotiation process need to be ensured [56], including the disclosure of information on medicines. Finally, a multi-level health security strategy, such as medical insurance for major diseases [57] and medical assistance for low-income people, can be adopted [29].

The NDPN has been carried out annually in China since 2016, and several previous studies have evaluated the effect of the NDPN policy conducted in 2016 and 2017. To our knowledge, we are the first to analyse the 2018 NDPN policy and most of our findings were similar to those of previous studies. Huang et al. [11], after the 2016 NDPN policy was implemented, found that the utilization of two anticancer medicines increased and the average daily cost decreased. Zhu et al. [58] found that the availability of 18 negotiated medicines in 2017 was improved and the average daily cost was reduced. However, these two studies above had fewer outcome measures and no control group. Sun et al. [10] found that the utilization, total expenditure, and availability of three negotiated anticancer medicines increased. Fang et al. [12] discovered that the availability, utilization, and average daily cost of 15 negotiated anticancer medicines in 2017 increased, but a control group was lacking and the number of hospitals was small. Zhang et al. [7] found that the average daily cost and the expenditure of 15 negotiated medicines in 2017 decreased, and the utilization increased. The increase in utilization and availability in our study was much higher than in the previous studies, probably due to the new NHSA conducting the negotiations.

We also found that the lag time between the release and implementation of the negotiation policy was shortened compared to previous studies [7,13]. In the 2018 NDPN, the utilization and cost of the negotiated anticancer medicines changed immediately in the first month after the policy implementation, indicating that the local government was more successful in implementing it. The NDPN policy was optimized after 2018, including the implementation of medicine floor price measurement, the introduction of competitive negotiation, and the strengthening of communication between the government and pharmaceutical companies [59].

Price negotiation has also been adopted to reduce drug prices in other countries. Price negotiations practised in Germany and Switzerland addressed the high prices of anticancer drugs, while increasing prices were observed in the USA without price negotiations [60]. After the implementation of price negotiations in Germany, drug prices in the USA were higher than those in Germany, and the divergence in drug prices between the two countries increased [61]. We now found new evidence to support price negotiation, which could be an option for countries looking to reduce the price of anticancer drugs and improve accessibility. Of course, some prerequisites and supporting measures were needed to conduct drug price negotiations.

Our study has some limitations. First, we calculated the annual cost without considering the discontinuation of medicine in patients, which may lead to an overestimation of the annual cost. Second, the four medicines in the control group may not be sufficiently representative. Since fewer anticancer medicines were not included in the NRDL throughout the study period, the control and intervention groups did not have exactly the same targets and indications. Another limitation is that unlisted anticancer drugs had lower availability during the list-forming process, so our results may overestimate the realistic availability. However, to avoid the change of drug utilization due to epidemiology, we still choose anticancer drugs instead of non-cancer-related drugs as the control. Third, due to the lack of procurement data from community pharmacies, we only counted the availability at hospitals.

## CONCLUSIONS

We found that the NDPN policy conducted in 2018 reduced the cost of anticancer medicines and improved availability, utilization, and affordability. China's experience in NDPN provides a reference for other countries that want to improve the accessibility of anticancer medicines. However, more measures are still needed to increase availability and make innovative anticancer medicines more affordable.

## Additional material


Online Supplementary Document
