# Supplementary material for: Impact of the national drug price negotiation policy on the utilization, cost, and accessibility of anticancer medicines in China: A controlled interrupted time series study
Source: J Glob Health. 2022 Dec 17;12:11016. doi: 10.7189/jogh.12.11016 (PMC9758701; doi:10.7189/jogh.12.11016)
Supplement: Online Supplementary Document [file jogh-12-11016-s001.pdf]

ONLINE SUPPLEMENTARY DOCUMENT

Title: Impact of the national drug price negotiation policy on the utilization, cost, and accessibility of anticancer medicines in China: A controlled interrupted time series study

Authors: Lele Cai[1], Tiantian Tao[1], Hongtao Li[1], Zhuolin Zhang[1], Lingli Zhang[1]*, Xin Li[1,2,3]*

Table S1 Availability of 17 anticancer medicines across eastern, middle, and western regions before and after the NDPN policy

Generic name	Availability before NDPN (%)				Availability after NDPN (%)			
	East	Middle	West	Total	East	Middle	West	Total
Afatinib	0.85	0.39	1.55	0.87	37.18	28.02	31.61	33.88
Axitinib	0.68	0.39	0.00	0.48	22.58	22.96	17.62	21.75
Azacitidine	0.34	0.39	0.00	0.29	32.77	27.24	19.69	28.97
Anlotinib	2.55	3.50	1.04	2.50	54.84	49.42	47.15	52.07
Octreotide	9.68	4.67	4.66	7.51	24.96	21.01	14.51	22.04
Osimertinib	3.57	1.95	0.52	2.60	51.44	43.19	49.22	48.99
Crizotinib	1.87	1.95	1.55	1.83	38.37	31.91	35.75	36.28
Nilotinib	8.32	10.12	12.95	9.62	25.30	21.01	23.83	23.97
Pegaspargase	15.28	19.07	14.51	16.07	28.18	31.13	29.02	29.07
Pazopanib	0.85	1.17	0.00	0.77	19.35	14.79	8.29	16.17
Regorafenib	0.68	2.33	1.04	1.15	34.47	29.57	27.46	31.95
Ceritinib	0.00	0.00	0.00	0.00	18.51	14.79	11.92	16.36
Sunitinib	4.41	4.28	1.55	3.85	29.37	28.40	25.91	28.49
Vemurafenib	0.00	0.00	0.00	0.00	11.04	11.28	5.70	10.11
Cetuximab	15.45	6.61	5.18	11.36	44.31	34.24	32.64	39.65
Ibrutinib	1.19	1.95	0.00	1.15	29.37	24.90	21.24	26.76
Ixazomib	0.85	0.00	0.00	0.48	26.66	19.46	15.03	22.71
Mean (SD)	3.92 (5.13)	3.46 (4.86)	2.62 (4.47)	3.56 (4.72)	31.1 (11.55)	26.67 (9.88)	24.51 (12.36)	28.78 (11.13)

SD – standard deviation.

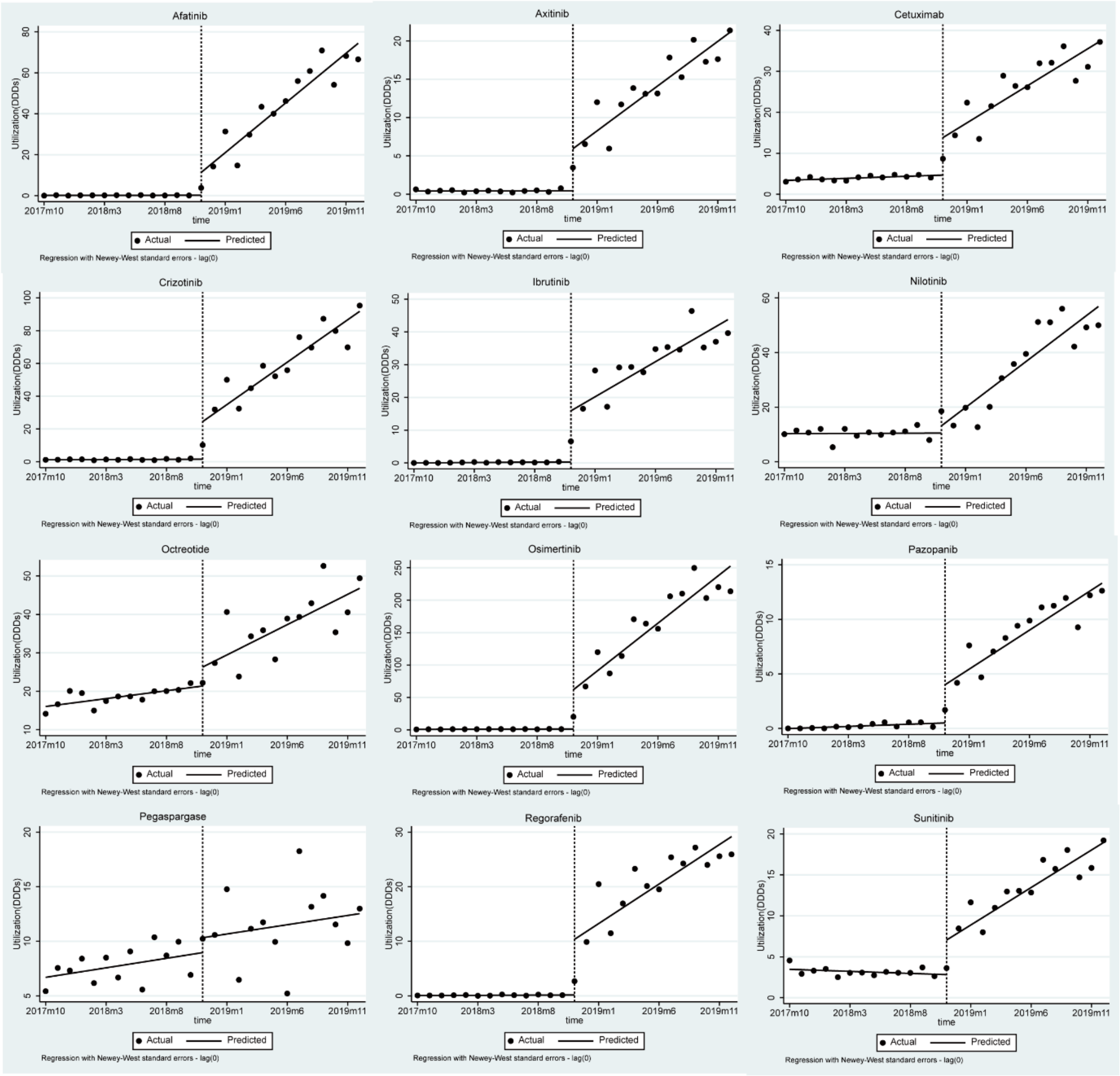

Figure S1 Changes in the observed and predicted utilization of 12 anticancer medicines in the intervention group.

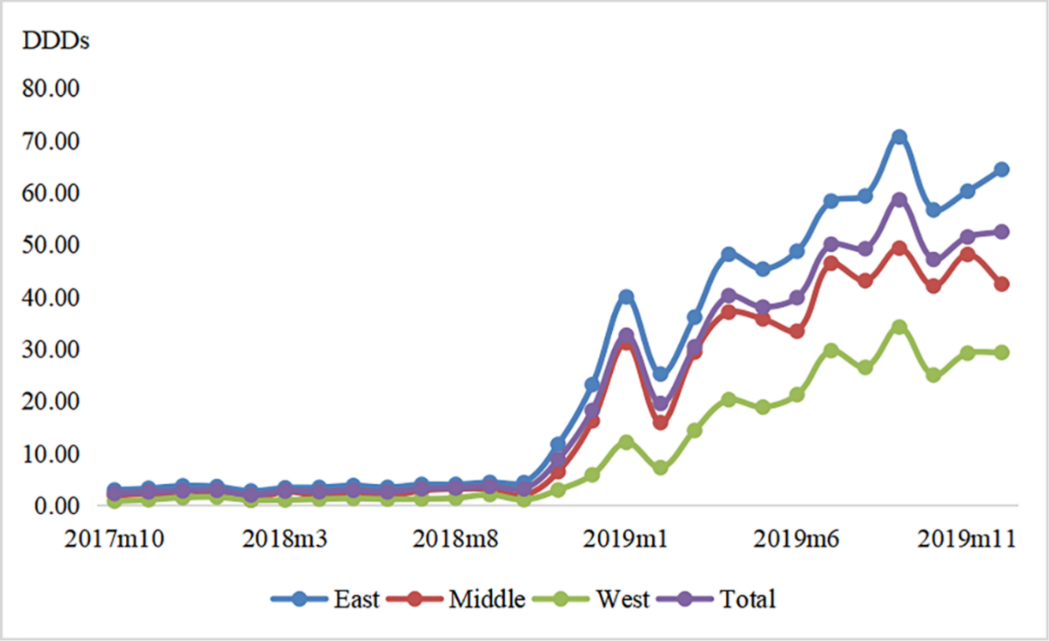

Figure S2 Changes in hospital average utilization of 17 anticancer medicines across eastern, middle, and western regions.

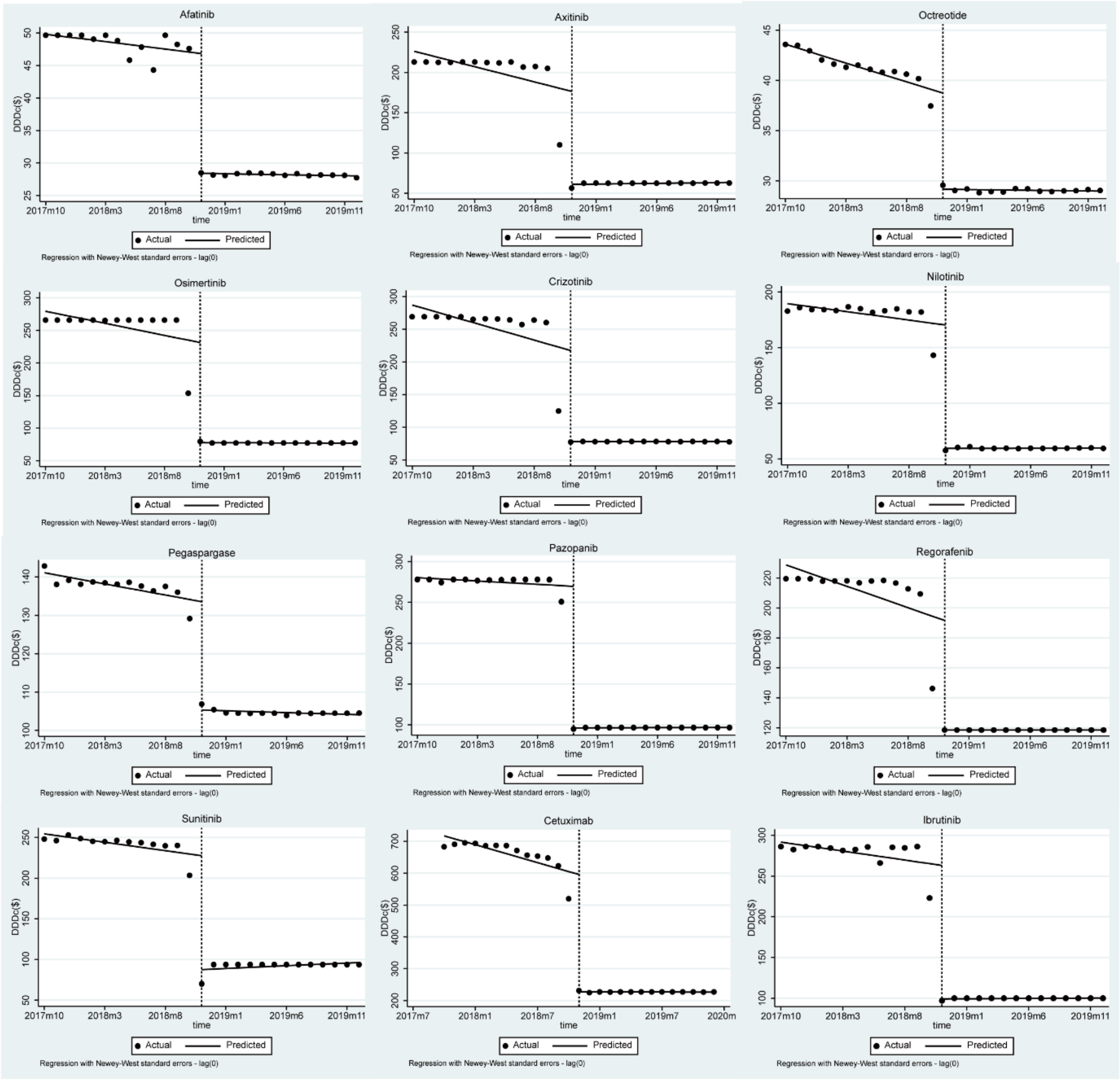

Figure S3 Changes in the observed and predicted DDDc of 12 anticancer medicines in the intervention group.
